# The muzzle to target distance —staining inside different parts of the firearm barrel

**DOI:** 10.1007/s00414-023-03141-8

**Published:** 2023-12-13

**Authors:** Christian Schyma, Rolf Müller, Julia Brünig, Martin Zieger, Silvia Utz, Melanie Grabmüller

**Affiliations:** 1 Institute of Forensic Medicine of the University of Bern, Murtenstrasse 26, 3008 Bern, Switzerland; 2 Criminal Investigation Service, Cantonal Police Department of Bern, Nordring 30, 3013 Bern, Switzerland; 3 Institute of Legal Medicine, University Bonn, Stiftsplatz 12, 53111 Bonn, Germany

**Keywords:** Firearms, Muzzle gas, Shot range, Biological traces, Molecular genetics, miRNA

## Abstract

Biological traces inside firearm barrels were observed as a result of contact shots to the head. The present study was conducted to investigate the influence of the muzzle to target distance on staining inside the anterior and posterior part of firearm barrels. Ninety-nine shots were fired to so-called reference cubes (10% gelatine, 12 cm edge length, embedded paint-blood-pad) using three current handguns. Shot range was varied from contact to 50 cm distance. High-speed cameras recorded external backspatter. Endoscopic examination assessed visible staining along the barrel. Each two swabbings were gathered from the anterior and the posterior part of the barrel. The first swabs were submitted to quantitative PCR, the second ones to DNA-RNA-co-extraction. Thorough mechanical and chemical cleaning was performed to avoid any contamination which was controlled by negative zero swabs after each cleaning. In single shots up to 50 cm distance, minimal, but DNA-positive sporadic traces were detected inside the barrel in vicinity of the muzzle. Visible complex staining varying in extent was observed in the anterior barrel part for 10 cm or less distance in dependence of the calibre. The posterior part showed detectable traces only after close range shots (< 5 cm). Generally staining inside the barrel decreased from the muzzle to the rear end, which correlated with the yield of DNA. Some contact shots did not cause any staining in the posterior part of the barrel despite massive external backspatter. Blood-specific miRNA was primarily found where DNA was detected. This experience encourages to take a second swab for RNA analysis. The amount of nucleic acids in the barrel at varying muzzle to target distances is subject to large variations between individual shots and therefore appears not suitable for a reliable determination of the shot distance in a particular case on its own. Instead, shot range estimation should also take into account morphology and distribution of traces inside the barrel.

**Supplementary Information:**

The online version contains supplementary material available at 10.1007/s00414-023-03141-8.

## Introduction

Gunshot injuries are a current topic in forensic routine. The most important question is to differentiate between homicide and suicide. This issue can only be resolved by a multidisciplinary strategy [1]. The unconditional cooperation of forensic pathologists, crime scene investigators, forensic scientists and police investigators is imperative. One aspect in the reconstruction of firearm related incidents — among many others — is the estimation of the shot range. While forensic pathologists examine the entry wound for signs of muzzle gas pressure, burns, darkening by soot or powder tattooing [2, 3], forensic scientists search for gunshot residues (GSR) as a marker for a relatively close distance which could be estimated by performing test shooting with the incidental gun and ammunition [4]. For decades, the firearm was regarded as an exclusive domain of the firearm identification service. However, Weimann described a homicide case, which occurred in 1922. The incidental gun was identified by Brüning who detected fat and protein containing material inside the barrel [5]. In 1934, Brüning and Wiethold published observations on tissue debris and blood inside firearm barrels after suicidal contact shots [6]. This phenomenon was repeatedly confirmed [7–10] and called the drawback effect [11]. McDonell and Brooks went beyond the simple distinction of blood-positive and blood-negative barrels measuring in which depth of the barrel blood was detected [11]. They saw a “relationship between discharge distance and the distance to which blood is drawn back into the barrel” [11]. Further investigation was based on the endoscopic examination of firearm barrels [12–16], which confirmed the presence of biological staining in many, but not all firearm barrels after contact shot to the head. To understand the phenomenon and its variability, systematic experimental research was conducted [17, 18] and a simple, but reliable target model [19] was developed which allowed for generating staining inside firearm barrels as well as observing the temporary cavity [20, 21] and backspatter [22]. Other models, like a Synbone® polyurethane skull, were proposed [23, 24]. However, the more complex the model, the more doubtful/difficult is the reproducibility of a physical experiment [25]. Euteneuer et al. reported on a series of experimental shots from various distances (0–50 cm) to the anatomical skull model [26]. The presence of “backspatter” inside the pistol and revolver barrel was assessed by quantitative PCR of swabs gathered from inside the barrel. Apart from the results of contact shots, their conclusion was sobering. A relationship between shooting distance and DNA yield could not be established [26].

The present study was conducted to clarify the influence of the muzzle-to-target distance on staining inside different parts of the firearm barrel. Strictly standardized experimental conditions comprised optimized observation by high-speed cameras, morphological assessment of traces using endoscopy and a differentiated sampling method.

## Material and methods

### Target models, firearms and ammunition

Target models were prepared as 10% gelatine reference cubes of 12 cm edge length [19]. A thin foil bag beneath the covering absorbent wipe contained a mixture of 2 ml heparinized blood, 2 ml acrylic paint (CPM, Erkrath, Germany) and 1 ml radiocontrast agent Micropaque® (Guerbet, Brussels, Belgium) [17]. Blood was taken by venipuncture from two informed and consenting adult volunteers (according to the approval by the ethics committee of the University Hospital Bonn). For each shooting series, half of the target models contained blood from donor A mixtured with red paint respectively donor B with blue paint. The differently marked models were alternated, when shooting was performed. Current handguns with four inch barrel length were chosen. Only non-deforming full metal jacketed bullets (FMJ) were fired. Firearms and ammunition are listed in Table 1.

**Table 1:** Firearms and ammunition

Firearm	Calibre	Type of firearm	Cartridge	Bullet weight		Barrel length [mm]
				[g]	[gr]	
Walther PP	7.65 mm Browning^1^	Semi-automatic pistol	7.65 mm FMJ S&B	4.8	73	98
Astra Cadix	.38 special	Revolver	.38 special FMJ UMC	8.4	130	100
Glock 19	9 mm Luger	Semi-automatic pistol	9 mm Luger FMJ S&B	8.0	124	102
Smith & Wesson	9 mm Luger	Semi-automatic pistol	9 mm Luger FMJ S&B	8.0	124	102

*FMJ* full metal jacketed bullet,* S&B* Sellier & Bellot (Vlašim, Czech Republic), *UMC* Union Metallic Cartridge Company (Remington, Lonoke, USA).

^1^.32 auto

### Shooting and high-speed video documentation

The experiments were performed in the indoor shooting range of the Criminal Investigation Service of the Cantonal Police Bern. Each three shots were fired per firearm and distance (*n* = 81). The muzzle to target distance decreased in following steps: 50 cm, 30 cm, 20 cm, 10 cm, 5 cm, 3 cm, 2 cm, 1 cm, 0 cm (contact). Additionally, close range distances *(n* = 18) were investigated in 0.5 cm steps (0 cm, 1 cm, 1.5 cm, 2 cm, 2.5 cm, 3 cm).

Two synchronized Fastcam SA-X2 (Photron Europe Ltd., West Wycombe, UK) recorded the shots with 40,000 frames per second and 10 µs exposure time. One camera was placed orthogonally to the line of fire, the other was in oblique position focussing the muzzle, where the muzzle to target distance allowed for it (10–50 cm).

### Assessment of staining inside the barrels and sampling procedure

All firearms were carefully cleaned as published [1]: after thorough mechanical cleaning using barrel cleaners of woollen felt (VFG, Giengen, Germany) soaked with Ballistol® (Klever GmbH, Aham, Germany) the barrel was filled with bleach (2.6% sodium hypochlorite) for 2 min. Then a new clean woollen felt was pulled through to dry the barrel. The successful result was checked by endoscopy using a Hawkeye borescope (Gradient Lens Corporation, Rochester, New York) with 0°-view optic. Finally, the inner surface of the barrel was wiped off using DNA-free forensic Swabs (Sarstedt, Nümbrecht, Germany) moistened with sterile desalted water (zero probe).

After each shot the outer surfaces of the gun were roughly cleaned. Then the external surroundings of the firearm’s muzzle were carefully cleaned with bleach. Video-endoscopy was performed before and after the sampling procedure. One after another two swabs were introduced from the muzzle wiping off the anterior half of the barrel. In analogous way, two other swabs were gathered from the posterior half accessing from the rear end of the barrel [15, 17–19].

Contamination was systematically avoided. All procedures were performed wearing surgical mask and gloves. Gloves were changed after each step of the procedure. Any contact of the swabs with outer surfaces of the firearm was strictly avoided. All working surfaces as well as the endoscope were cleaned after each sampling procedure.

### Molecular genetic analyses

All the samples were blinded and independently analysed in two molecular genetic laboratories. DNA concentration was analysed each in the first swab from the anterior and the posterior part. The second pair of swabs was submitted to RNA-DNA-co-extraction.

Laboratory Bern performed an automated DNA extraction of samples in 500 µl lysis buffer from the PrepFiler Express™ kit (Thermo Fisher, USA) and 5 µl 1 M DTT (Merck, Germany). Samples were shook on a Precellys24® homogenizer (Bertin Instruments, France) 2 × 30 s at 5900 rpm and centrifuged for 2 min at 13,000 rpm on an Eppendorf MiniSpin®, followed by 40 min incubation at 70 °C and 750 rpm. After 18 additional hours at 56 °C and 400 rpm, the swab heads were transferred to a spin basket provided with the PrepFiler Express™ kit and centrifuged for 3 min at 13,000 rpm on an Eppendorf MiniSpin® to recover the lysis solution. DNA from the lysis solution was extracted with the AutoMateExpress™ device (Thermo Fisher, USA) and an elution volume of 50 µl. DNA quantification was done by real-time PCR (qPCR) using the Quantifiler® HP DNA Quantification kit on a 7500 RT PCR System (both Thermo Fisher, USA).

Laboratory Bonn performed a manually RNA-DNA-co-extraction as previously published [1]. RNA contamination was prevented by cleaning all surfaces, instruments and devices using RNase-Zap® (Ambion, Austin, TX, USA) and Roti®-Nukleinsäurefrei (Carl Roth, Karlsruhe, Germany) as well as RNase-free reagents and plastic consumables. RNA extraction, quantification and integrity assessment [27] were performed as previously published [28, 29]. Co-extraction of DNA was performed by diverting 20 μl of the RNA lysate containing DNA to the DNA extraction procedure of the PrepFiler® Forensic DNA Extraction Kit (Thermo Fisher, USA) processing it according to manufacturer’s instructions. DNA concentration, degradation and the presence of PCR inhibitors were measured by quantitative PCR (qPCR) using the PowerQuant™ System (Promega, Mannheim, Germany) as recommended by the manufacturer on an ABI Prism® 7500 Sequence Detection System (Life Technologies).

The choice of blood- (miR-451a) and brain tissue-specific (miR-124a) miRNA as well as that of the reference gene miR-191 for qPCR data normalization has been already described [29] according to the MIQE-guidelines [30]. Reverse transcription and qPCR were performed according to the previously published procedure [29]. To calculate quantification cycle values and amplification efficiencies from raw data the LinRegPCR analysis program v2015.1 [31] was applied.

## Results

### Visual staining inside the barrels

**Fig. 1:**
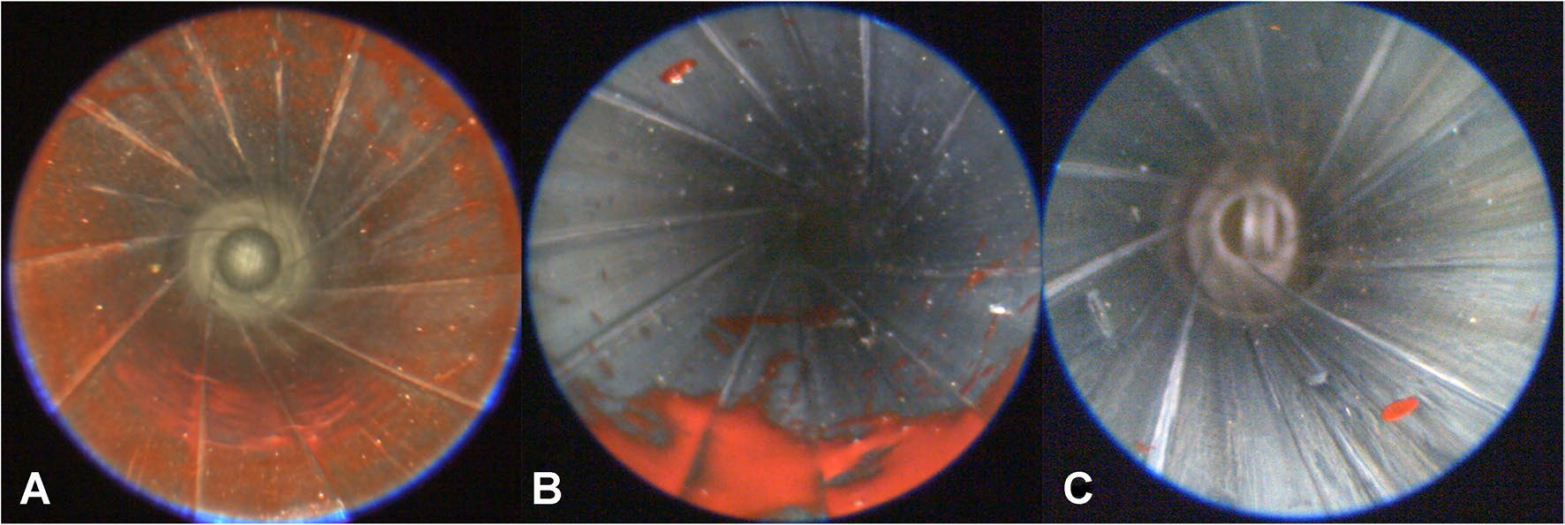
Endoscopic view of the 9 mm Luger Smith&Wesson barrel, captured just in front of the muzzle. **A** Contact shot. **B** Muzzle to target distance 1.5 cm. **C** Muzzle to target distance 3 cm

**Fig. 2:**
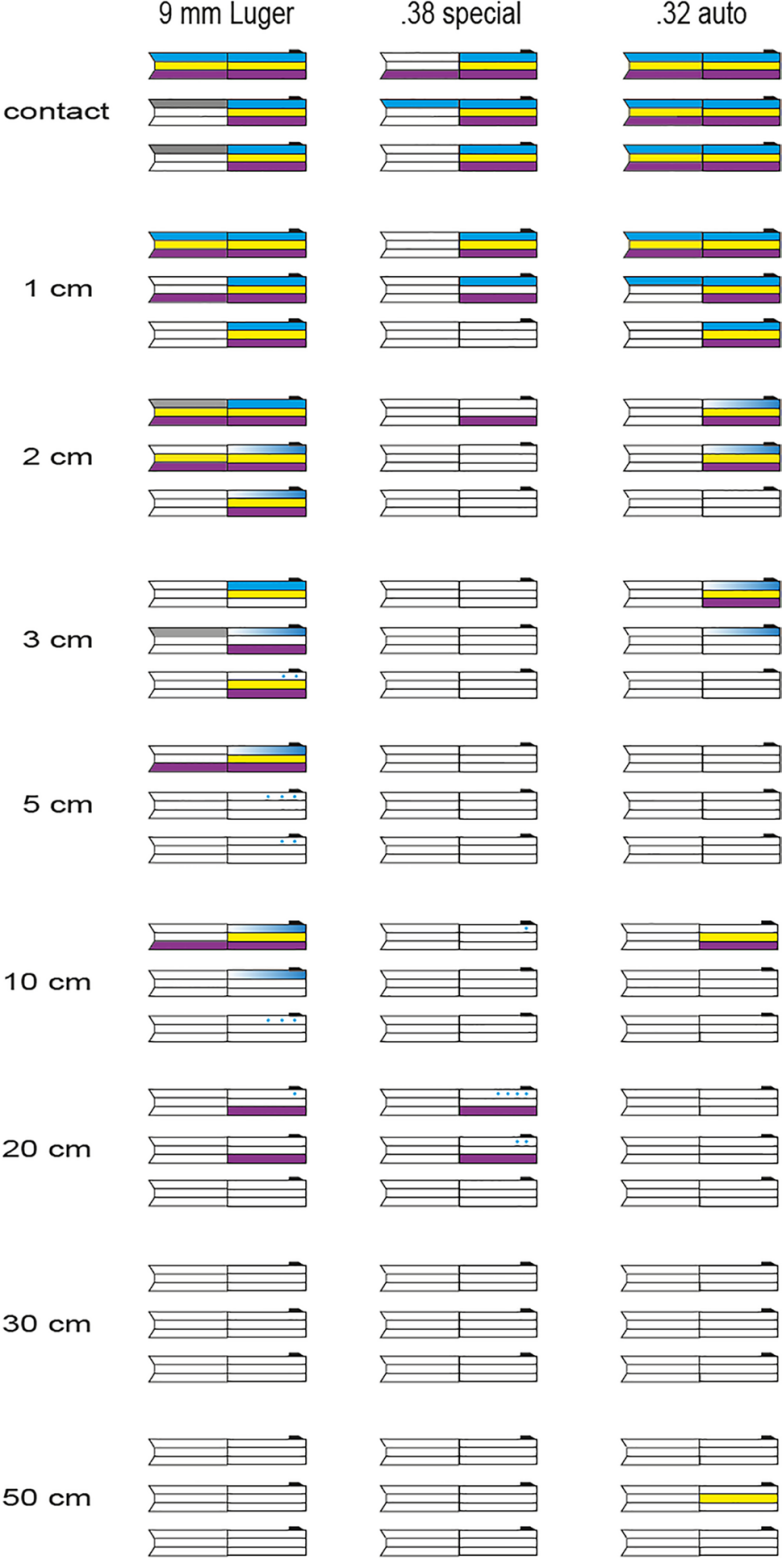
Graphical presentation of the results obtained with three different methods - endoscopy:  colored traces;  traces only in vicinity of the muzzle;  single spots;  dark traces. DNA  . RNA

Video endoscopy after each shot aimed to detect visible traces. Figure 2 displays the endoscopic results differentiated for the anterior and the posterior part of the barrel. Complex pattern (elongated, spatter-like, net like forms, stripes etc. (Figure 1A, B)) were summarized as staining (blue marks in Fig. 2). In 7 of 81 shots, single spots of acrylic paint were observed and counted (Figs. 1 C and 2). To simplify the reading, hereinafter all linear measures refer to the muzzle to target distance.

**Fig. 3:**
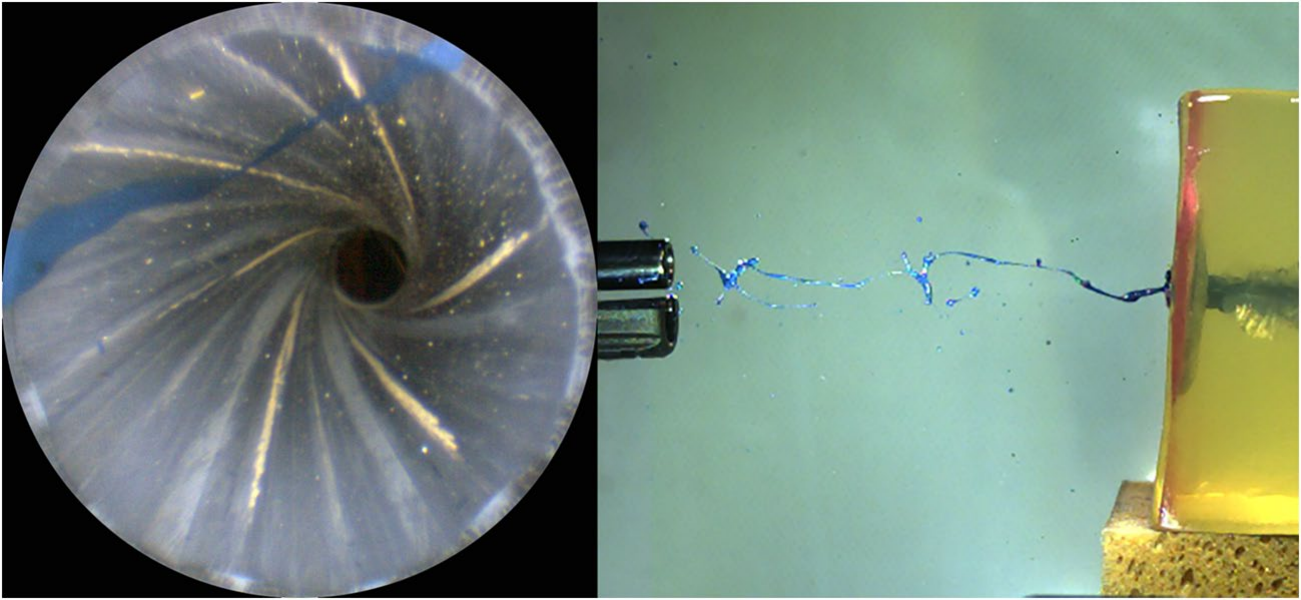
Endoscopic view captured just in front of the muzzle. Insight into the barrel of the Glock pistol cal. 9 mm Luger showing staining in vicinity of the muzzle caused by backspatter. The shot was fired from 10 cm distance. About 82 ms later, a linear jet with ramifications reached the muzzle

**Fig. 4:**
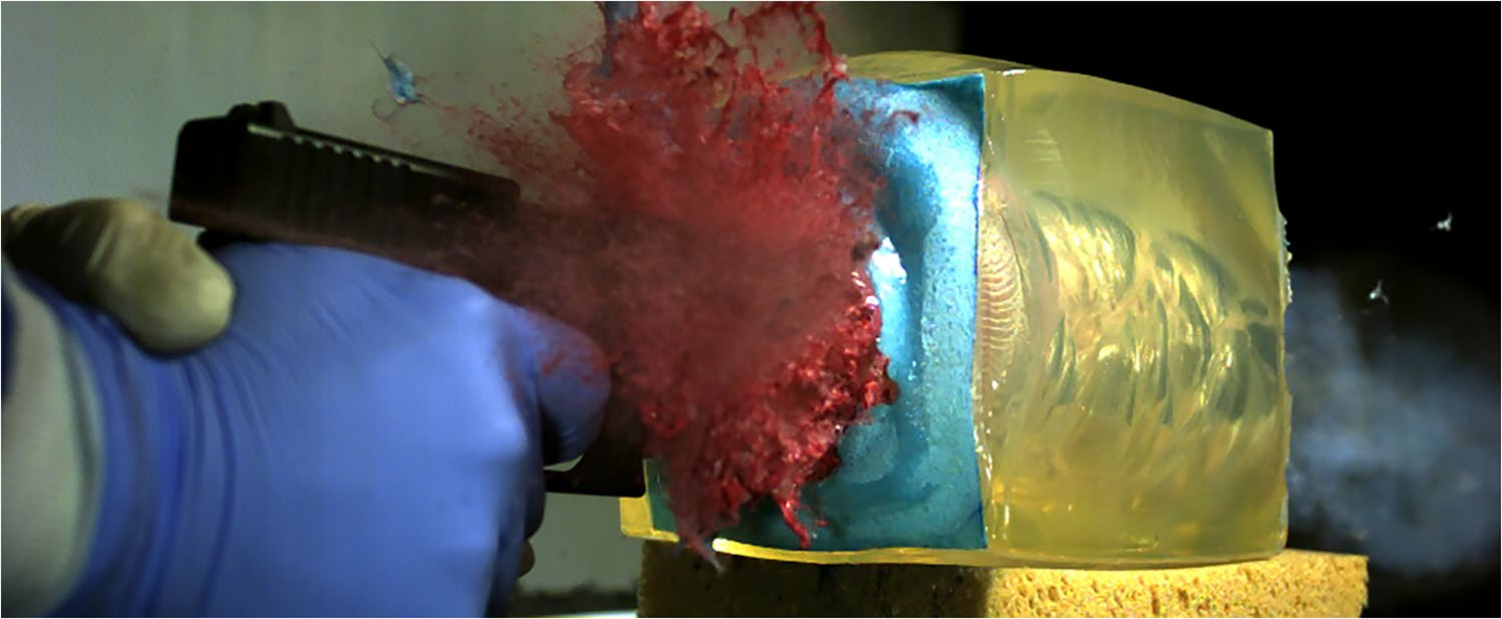
Contact shot using the 9 mm Luger Glock pistol. Despite massive external backspatter, no DNA was found in the posterior part of the barrel

The 9 mm Luger pistol showed staining in the anterior half of the barrel up to 3 cm (one of three shots). From 3 to 10 cm, much less traces were found inside the barrel. They were located exclusively in vicinity of the muzzle (light blue marks in Fig. 2). High-speed records showed backspatter, from the target model ejected fluid reaching the muzzle (Figs. 3 and 4). In one shot from 20 cm distance, only a single spot was found next to the muzzle. Larger shot ranges did not produce staining. The posterior part of the 9 mm Luger barrel showed staining in all contact shots and in each one of three shots up to 3 cm. Some marks in Fig. 2 are grey and indicate a visible dark staining without identifiable color. This classification was admissible because the control endoscopy before the shot had shown a clean barrel without discoloration. Gelatine spatters might be the origin of such traces.

The most impressive load of traces was observed in contact shots using the .32 auto pistol. In all three shots staining reached the chamber. The anterior part showed staining in all three 1 cm shots. In two of them, also distinct staining was observed in the posterior part. Shots from 2 to 3 cm distance just produced traces in vicinity of the muzzle (light blue marks in Fig. 2).

In contrast to the semi-automatic pistols, the revolver cal. .38 special exhibited a rather poor result of visible traces. Contact shots and 1 cm shots led to staining in the anterior half of the barrel, whereas staining in the posterior half was only observed in one of three contact shots. However, single spots were observed near the muzzle in two shots from 20 cm distance.

For the anterior part of the barrel, the results of the different firearms were rather similar. Staining in the posterior part was limited for 9 mm Luger to maximum 3 cm distance, for .32 auto pistol to 1 cm shot range and for the revolver to contact shots. A previous study showed similar distances for effects of muzzle gases on the temporary cavity [21].

Additional close range shots (Table 2) in cal. 9 mm Luger produced in some of the shots distinctive staining in the posterior part. Also, for calibre .32 auto, the visual results in the posterior part were heterogeneous. The posterior part of the .38 special revolver’s barrel was endoscopically nearly negative. Only after two contact shots, few traces were observed.

**Table 2:** Additional close range shots *(n* = 18)

	9 mm Luger		.38 special		.32 auto	
Distance [cm]	Part of the barrel		Part of the barrel		Part of the barrel	
	Anterior	Posterior	Anterior	Posterior	Anterior	Posterior
3.0	**0.11**	–	–	–	**0.40**	**–**
2.5	**18.64**	0.15	–	–	**2.70**	**0.26**
2.0	**39.16**	0.90	**2.93**	–	**35.05**	**0.43**
1.5	**41.36**	3.61	**1.59**	–	**31.05**	0.41
1.0	**27.70**	**2.94**	**6.92**	–	**29.30**	0.07
Contact	**13.70**	**2.19**	**3.97**	**0.16**	**25.15**	0.18

DNA yield in ng. Endoscopically visible traces are marked bold. [ - ] indicates no DNA detected

### DNA results after PCR of the swabs

All zero probes, in both laboratories, were DNA negative. No contamination was detected, even though external backspatter and staining inside the barrel next to the muzzle was important after close range shots. Figure 2 gives a qualitative overview of molecular genetic findings. Results of quantitative PCR are given in Table S1. Generally, shooting distances over 10 cm led to negative DNA results. However, a swab from the anterior barrel part of the .32 auto pistol after a shot range of 50 cm contained 0.40 ng DNA. Endoscopy of the barrel had been negative. The first hypothesis of a contamination could be excluded, because high-speed video had captured a tiny droplet while entering the muzzle. In dependence of the calibre, the anterior part of the muzzle was mostly positive up to 3 cm distance. Only eight shots—exclusively contact or close range shots — provoked measurable results in the posterior barrel part, but the DNA concentrations were 10 to 100 times less than in the anterior part for the same shot (Table S1). A total of 11 of 18 additionally performed close range shots led to staining in the posterior barrel part with DNA yield from 0.07 to 3.61 ng (Table 2). The .38 special revolver showed the poorest result: only in one of four contact shots, DNA was found in the posterior barrel part. Already, shots with few centimetres muzzle-target distance (≥ 1 cm) did not produce DNA containing staining in the posterior part of the revolver barrel.

A comparison of endoscopic assessment and molecular genetic analysis showed that both approaches matched very well, but some swabs contained DNA, whereas no visible staining had been observed during endoscopy (Table 2). The inverse constellation, visible staining without DNA yield, also occurred in few cases.

### RNA results obtained by RNA-DNA-co-extraction

The anterior barrel part was found RNA positive up to 20 cm distance (Fig. 2). This result was in part independent of the presence of DNA (9 mm Luger, .38 special). After close contact shots, positive DNA detection was almost always combined with positive findings of blood-specific miRNA. In the posterior part, most of the RNA-positive results were obtained after close contact shots using the 9 mm Luger pistol and contact shots using the .32 auto Walther pistol. In contrast, the .38 special revolver only led in one of three contact shots to detection of RNA, all other experimental shots remained negative for RNA.

## Discussion

In medico-legal investigation of deaths by firearms, the firearm was long time exclusively examined by the criminal investigation service. However, the presence of blood [8–10] or tissue debris inside the barrel implied a relation to the victim. The ongoing progress in molecular genetics enabled the identification of the victim using PCR [14, 15, 32]. Biological traces were primarily associated with contact shots, most of them suicides and shots to the head [14, 15]. Of course, the question arose if staining inside firearm barrels was exclusive to contact shots. Backspatter — biological material of the entry wound ejected against the direction of fire — was known to cover large areas and distances of several meters. Logically, Courts et al. asked “How far does it get ?” [33]. Experimental investigation was continued using skull models focussing on backspatter after various shot ranges up to 50 cm. The authors summarized that no meaningful correlation was found between DNA yield, backspatter distribution and shooting distance [26]. This conclusion was based on an extended sampling on the outside of the firearm and the inside of the barrel. However, neither endoscopic examination nor differentiated sampling inside the barrel had been performed. The separate sampling of traces from the anterior and the posterior part of the barrel’s inner surface has already been proposed for experimental ballistics [34] as well for real case work [15].

About hundred shots were fired on standardized target models [19] and recorded with high-speed cameras. The assessment of staining inside the firearm barrel was performed by endoscopy [16] and parallel DNA and RNA analysis. The systematic experimental investigation of staining inside firearm barrels using a combined visual (acrylic paint) and molecular genetic (human blood) approach revealed an obvious difference between shot ranges above 10 cm and close range shots. Single spots of paint were observed inside the barrel in vicinity of the muzzle, even when the muzzle to target distance was 10 cm or greater. In some experimental shots, it was possible to observe the formation of the trace as a consequence of backspatter recorded by high-speed video (Fig. 3). Previous research had demonstrated a linear ejection of fluid after shots from 5 cm distance and more [22]. If such a jet is meeting the muzzle of a gun, it depends on the movement of the gun, the muzzle-to-target distance and the velocity of the jet. A shot fired by a Walther pistol from 50 cm distance led to such a match: after the bullet had left the barrel, the muzzle moved quickly up and returned slowly (128 ms after) roughly in its previous position when a little droplet of paint disappeared in the muzzle. RNA analysis might be useful to detect such sporadic staining, however the effectivity seemed not convincing. Only in two shots (20 cm distance, 9 mm Luger, and .38 special) blood-specific miRNA was detected as sole biological trace.

The most important finding of the study was the significant difference of staining in the anterior and the posterior part of the barrel. Only shots from 3 cm distance or less generated visible traces in the posterior part, albeit not in all cases. Contact shots, especially performed with semi-automatic pistols, provoked in part abundant staining. However, endoscopy reached its optical limits when the spray of acrylic paint was too fine or the contrast of color was too low in the bluing barrels. The visual impairment by corrosion of the barrel due to excessive use of bleach [1] was another limiting factor. Even more, the expectations were high that PCR would overcome this issue. Actually, in six close range shots, DNA was successfully amplified, where the endoscopic examination was negative. RNA contributed only few additional information. Mostly, RNA was positive when DNA was found. Still, it is reminded that RNA-extraction was performed from the second swab while the first swab was used for DNA-analysis. From the forensic point of view, this might be an encouragement that a second swab contains enough miRNA to identify body fluid or tissue.

The study showed another forensically important aspect. Contact shots generated mostly detectable staining in the posterior part of the barrel, but not in all experimental shots. Each two contact shots using the 9 mm Luger pistol (Fig. 4) and the .38 special revolver were negative in molecular genetic analysis of the swabs gathered in the posterior barrel part. The evaluation of the high-speed records did not reveal a difference between these “negative” and corresponding staining-positive results. The development of the temporary cavity as well as external backspatter were similar. In return, it can be derived that massive external backspatter (Fig. 4) does not automatically lead to detectable staining in the posterior part of the barrel. The influence of muzzle gas pressure on the temporary cavity [21] and the ejection of fluid against the line of fire [22] has been demonstrated previously. The mechanism of staining in the depth of the firearm barrel, far from the muzzle remains yet unclear.

Three current handguns in forensically relevant calibres were used to perform test shootings. Distant shots (> 10 cm) did not show differences in staining inside the barrel. Though, close range shots indicated a different effect of the two semi-automatic pistols and the revolver, especially concerning staining in the posterior barrel part. The 9 mm Luger pistol — 490 J mean kinetic energy of the bullet — is certainly not comparable to the two other calibres. However, the .38 special revolver and the .32 auto pistol delivered similar kinetic energies of about 230 J [35]. Their significantly different staining in the posterior part of the barrel might be an indication that the system of the weapon — revolver or pistol — plays a role in the formation of traces inside the barrel.

Finally, the meaning of DNA amount in the context of staining inside firearm barrels has to be addressed. The yield of DNA was subject to large variations, rendering it challenging to assume a reliable relation between the recovered DNA amount and the muzzle to target distance. This observation confirms the results of the working group of Courts [26]. Nevertheless, the DNA yield was always higher in the anterior part of the barrel than in the posterior, which correlated with endoscopic findings and confirmed previous results [19]. This observation of a gradual distribution is calling for a “mapping” of the staining inside firearm barrels. A morphological detected biological trace, localized in a distinct part of the barrel might have more evidence than blind sampling alone.

## Conclusions

• External backspatter can cause traces inside the barrel in vicinity of the muzzle, even in distant shots.

• Close range shots cause staining which decreases from the anterior to the posterior part of the barrel.

• Experimental staining in the posterior barrel part was only observed in close range shots (< 5 cm distance).

• Experimental contact shots did not imperatively provoke staining in the posterior barrel part.

• Shot range estimation based exclusively on DNA results is not advisable.

### Supplementary Information

Below is the link to the electronic supplementary material.

Supplementary file1 (DOCX 17.1 KB)

## Declarations

The authors ensure that the work described has been carried out in accordance with The Code of Ethics of the World Medical Association (Declaration of Helsinki) for experiments involving humans. The study was approved by the ethics committee of the University Hospital Bonn.
