# Supplementary material for: The muzzle to target distance —staining inside different parts of the firearm barrel
Source: Int J Legal Med. 2023 Dec 13;138(3):1149–56. doi: 10.1007/s00414-023-03141-8 (PMC11003904; doi:10.1007/s00414-023-03141-8)
Supplement: Supplementary file 1 — Supplementary file1 (DOCX 17.1 KB) [file 414_2023_3141_MOESM1_ESM.docx]

**Table S1** - Results of quantitative PCR after 81 shots

Only eight shots led to DNA positive results in the posterior part of the barrel (contact or close range shots). DNA yield is displayed in ng.

| **9 mm Luger** |  | | | | | |
| --- | --- | --- | --- | --- | --- | --- |
| Distance [cm] | Part of the barrel | | Part of the barrel | | Part of the barrel | |
|  | Anterior | Posterior | Anterior | Posterior | Anterior | Posterior |
| 50 | ̶ | ̶ | ̶ | ̶ | ̶ | ̶ |
| 30 | ̶ | ̶ | ̶ | ̶ | ̶ | ̶ |
| 20 | ̶ | ̶ | ̶ | ̶ | ̶ | ̶ |
| 10 | 0.06 | ̶ | ̶ | ̶ | ̶ | ̶ |
| 5 | ̶ | ̶ | ̶ | ̶ | 12.77 | ̶ |
| 3 | 0.44 | ̶ | 0.08 | ̶ | ̶ | ̶ |
| 2 | 27.58 | 0.08 | 4.21 | 1.81 | 0.17 | ̶ |
| 1 | 0.98 | ̶ | 0.47 | ̶ | 78.70 | 5.62 |
| Contact | 0.42 | ̶ | 3.35 | 0.09 | 1.27 | ̶ |
|  |  |  |  |  |  |  |
| **.38 special** |  | | | | | |
| Distance [cm] | Part of the barrel | | Part of the barrel | | Part of the barrel | |
|  | Anterior | Posterior | Anterior | Posterior | Anterior | Posterior |
| 50 | ̶ | ̶ | ̶ | ̶ | ̶ | ̶ |
| 30 | ̶ | ̶ | ̶ | ̶ | ̶ | ̶ |
| 20 | ̶ | ̶ | ̶ | ̶ | ̶ | ̶ |
| 10 | ̶ | ̶ | ̶ | ̶ | ̶ | ̶ |
| 5 | ̶ | ̶ | ̶ | ̶ | ̶ | ̶ |
| 3 | ̶ | ̶ | ̶ | ̶ | ̶ | ̶ |
| 2 | ̶ | ̶ | ̶ | ̶ | ̶ | ̶ |
| 1 | ̶ | ̶ | ̶ | ̶ | 0.16 | ̶ |
| Contact | 18.23 | ̶ | 18.44 | ̶ | 14.47 | ̶ |
|  |  |  |  |  |  |  |
| **.32 auto** |  | | | | | |
| Distance [cm] | Part of the barrel | | Part of the barrel | | Part of the barrel | |
|  | Anterior | Posterior | Anterior | Posterior | Anterior | Posterior |
| 50 | ̶ | ̶ | 0.40 | ̶ | ̶ | ̶ |
| 30 | ̶ | ̶ | ̶ | ̶ | ̶ | ̶ |
| 20 | ̶ | ̶ | ̶ | ̶ | ̶ | ̶ |
| 10 | 3.38 | ̶ | ̶ | ̶ | ̶ | ̶ |
| 5 | ̶ | ̶ | ̶ | ̶ | ̶ | ̶ |
| 3 | ̶ | ̶ | ̶ | ̶ | 1.89 | ̶ |
| 2 | 0.73 | ̶ | ̶ | ̶ | 1.98 | ̶ |
| 1 | 40.43 | ̶ | 20.12 | 0.13 | ̶ | ̶ |
| Contact | 65.60 | 0.17 | 35.03 | 0.12 | 89.78 | 5.38 |

[-] no DNA detected
